# Toxin-Pathogen Synergy Reshaping Detoxification and Antioxidant Defense Mechanism of *Oligonychus afrasiaticus* (McGregor)

**DOI:** 10.3390/molecules23081978

**Published:** 2018-08-08

**Authors:** Ahmed Mohammed AlJabr, Abid Hussain, Muhammad Rizwan-ul-haq

**Affiliations:** Laboratory of Bio-Control and Molecular Biology, Department of Arid Land Agriculture, College of Agricultural and Food Sciences, King Faisal University, Hofuf 31982, Al-Ahsa, Saudi Arabia; aljabr@kfu.edu.sa (A.M.A.); mianrizwan15@gmail.com (M.R.-u.-h.)

**Keywords:** compatibility, synergism, antagonism, biological control, phytol, *Beauveria bassiana*, host defense, plant secondary metabolite, old world date mites

## Abstract

Current study reveals the likelihood to use pathogen and toxin mutually as an effective and eco-friendly strategy for *Oligonychus afrasiaticus* (McGregor) management, which could reduce toxicant dose and host killing time. Therefore, phytol and *Beauveria bassiana* in different proportions were evaluated to determine their effectiveness. Prior to ascertaining host mortality and defense mechanisms, we have recorded in vitro action of phytol using different concentrations (0.70, 1.40, 2.10, 2.80, and 3.50 mg/mL) against *B. bassiana* suspension. In vitro compatibility assays revealed that growth parameters (vegetative growth, sporulation, and viability) of *B. bassiana* were least affected by the action of phytol at all tested concentrations. Biological Index of *B. bassiana* exhibited compatibility with phytol allowed us to conduct Joint toxicity bioassays in which phytol and spores mixed in different proportions in order to attain maximum treatment effect in terms of high mortality at low concentration under short time. Results revealed that joint-application exhibited both synergistic (treatments with higher proportions of phytol), and antagonistic interaction (treatments with higher proportions of spores) interactions. Biochemical mechanisms involved in host antioxidant and detoxification response were explored by quantifying their respective enzymatic activities. Lethality of different treatments induced different patterns of detoxification enzymes including glutathione S-transferase (GST) and acetylcholinesterase (AchE). Overall, the least potent treatments (20% phytol:80% spores, and 40% phytol:60% spores) established in the current study induced relatively higher GST and AchE activities. On the other hand, the most potent treatment (80% phytol:20% spores) at its maximum concentration exhibited negligible relative GST and AchE activities. Antioxidant enzyme activities of CAT and SOD measured in the current study showed moderate to complex interaction might because of toxin-pathogen remarkable synergy. This study suggested that joint application of phytol with *B. bassiana* spores have shown tremendous acaricidal potential and found to be promising new strategy for controlling old world date mites.

## 1. Introduction

The date palm (*Phoenix dactylifera* L.) is the dominant component of the oasis ecosystem responsible for the opening of vast desert areas for human activities. The unique nutritional characteristics of date palm fruit, exceptional tolerance against drought, salinity and harsh temperature, religious, and social affiliation of date palm with Arab culture make it the most preferred palm species to be cultivated in the oasis ecosystem. The date palm is the major fruit crop of the Kingdom of Saudi Arabia harvested on 145,516 ha with production of 964,536 tonnes during the year 2016 [1]. Presently, yield of dates in Saudi Arabia tremendously declined (66,284 hg/ha) compared to twenty-eight years ago yield (80,161 hg/ha) [1]. The rational decrease in yield mainly attributed to the pest infestations on date palm and their fruit.

*Oligonychus afrasiaticus* (McGregor) is a widespread species of spider mites infesting the fruits of date palm in Middle East (Saudi Arabia [2], Oman [3], Iraq [4], Iran [5], Egypt [6], Israel [7], Yemen [8]), and North Africa (Tunisia [9], Algeria [10]). The infestation of old world date mites starts after fruit set and multiply logarithmically on the *kimri* stage of the fruit by spinning silken webs around date bunches [11]. Fine dust particles and exuviae stuck in their silken web making a favorable environment for the development and reproduction of *O. afrasiaticus* populations. The infested immature greenish dates after attack turn to reddish-brown color with scabbed fruit appearance [12]. Such damages imparted significant reduction in fruit quality and quantity and making dates unfit for human consumption. Management of *O. afrasiaticus* infestations mainly relied on the frequent application of wide range of synthetic acaricides [7,13,14,15,16]. However, indiscriminate application of synthetic acaricides resulted undesirable scenarios due to residual toxicity, poor selectivity, environmental pollution, bioaccumulation, and low efficacy [13,17]. Thus, there is a dire need to focus on the development of alternate eco-friendly bio-control agents safe for humans with the least toxicity against non-target organisms.

The incorporation of fungal pathogens into the Integrated Pest Management program of *O. afrasiaticus* attained particular attention because of host specificity, persistence in target host population, numerous isolates and eco-friendly [18]. Entomopathogenic fungi especially isolates of *Beauveria bassiana* are well-known for their pathogenicity against different target pest species [19,20,21,22]. Spores of the isolates of entomopathogenic fungi impart mortality in the target pest by overcoming the complicated host defense related multiple mechanisms [18,23,24,25,26]. Therefore, spores of the isolates of entomopathogenic fungi require prolonged time period to impart significant mortality in target pest population [27]. Consequently, “dual attack” approach in which fungal pathogen along with bio-pesticide is simultaneously applied to reduce the dose of toxicant and reduce the host killing time of fungal pathogen [28,29]. In addition, joint application of toxicant and fungal isolate could easily overcome the host antioxidant defense related enzymes such as catalase (CAT) and superoxide dismutase (SOD) and detoxification related enzymes such as glutathione S-transferase (GST) and acetylcholinesterase (AChE) [21,30,31]. Previous study clearly described that host defense-related enzymes play crucial role by safeguarding the pest from toxins and pathogens. Furthermore, these defense related enzymes define the fate of target host against invading pathogens and toxicants. However, no data are available on the activities of defense related enzymes in *O. afrasiaticus* in response to invading fungal pathogen and/or toxicant.

The quest for the isolation and development of plant based secondary metabolites is the screening of plant extracts for the control of *O. afrasiaticus* populations. In this regard, we have tested the toxicity of crude extracts of different plant species against *O. afrasiaticus* in our preliminary study (Unpublished, data not shown). We found phytol the most toxic fraction isolated from the crude extract of *Cucumis sativus* against *O. afrasiaticus*. We undertook the current investigation to determine the feasibility of the joint action of spore suspension of *B. bassiana* with plant secondary metabolite, Phytol against the infestations of old world date mites. The integration of *B. bassiana* with phytol require multiple experimentation on different aspects (1) determining the pathogenicity of *B. bassiana* and toxicity of Phytol against old world date mites; (2) evaluating the spores viability, sporulation and vegetative growth in the presence of Phytol to know their compatibility; (3) utilizing the multiple concentrations of phytol and *B. bassiana* and their interaction to determine synergism among them; and (4) exploring the host detoxification and antioxidant defense mechanism in response to phytol and/or *B. bassiana*. Toxin-pathogen synergy experimentation will lay the foundation for the development of an eco-friendly alternative control strategy against the infestations of old world date mites.

## 2. Results

### 2.1. Compatibility of Beauveria bassiana B6903 with Phytol

**Figure 1:**
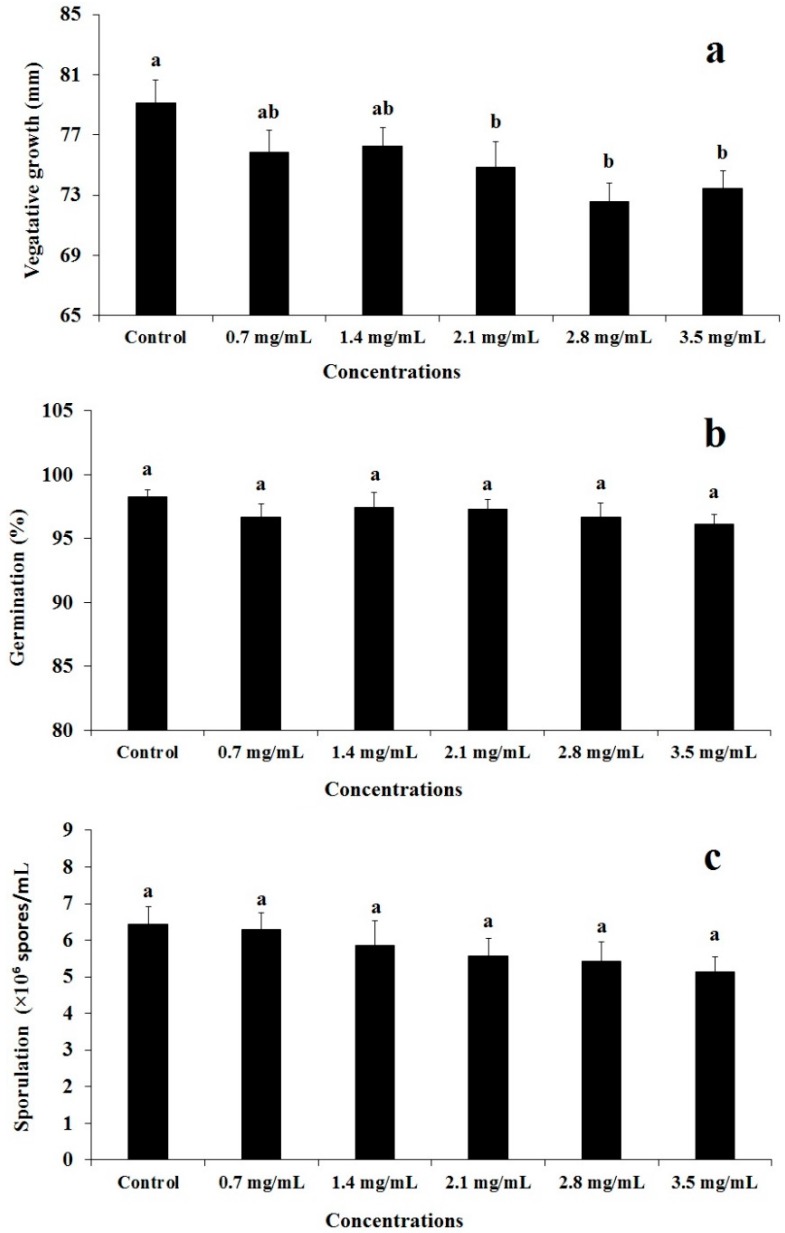
Effect of various concentrations of phytol prepared in DMSO on the vegetative growth (**a**), percent germination (**b**), and sporulation (**c**) of *B. bassiana* B6903. Values are the means of seven independent replicates. Bars with similar letter(s) are not significantly different (Fisher’s LSD test, α = 0.05).

The vegetative growth, sporulation, and percent germination of the spores of *B. bassiana* B6903 in the presence of different doses of phytol revealed dose-dependent response. The vegetative growth of *B. bassiana* B6903 that ranged from 73 mm to 80 mm showed significant differences (*F* = 2.74; df = 5, 36; *p* = 0.0337) among all tested doses of phytol (Figure 1a). Overall, dose-dependent vegetative growth reduction ranged from 3.61% to 8.30%. However, percent germination (*F* = 0.66; df = 5, 36; *p* = 0.6591), and sporulation (*F* = 0.95; df = 5, 36; *p* = 0.4584) revealed non-significant effect in response to different doses of phytol as shown in Figure 1b,c, respectively. The biological index (BI) values for all tested doses of phytol ranged from 87 to 97 as shown in Table 1. These results indicated that all tested concentrations of phytol are compatible with the spores of *B. bassiana* B6903.

### 2.2. Concentration Mortality Response

**Figure 2:**
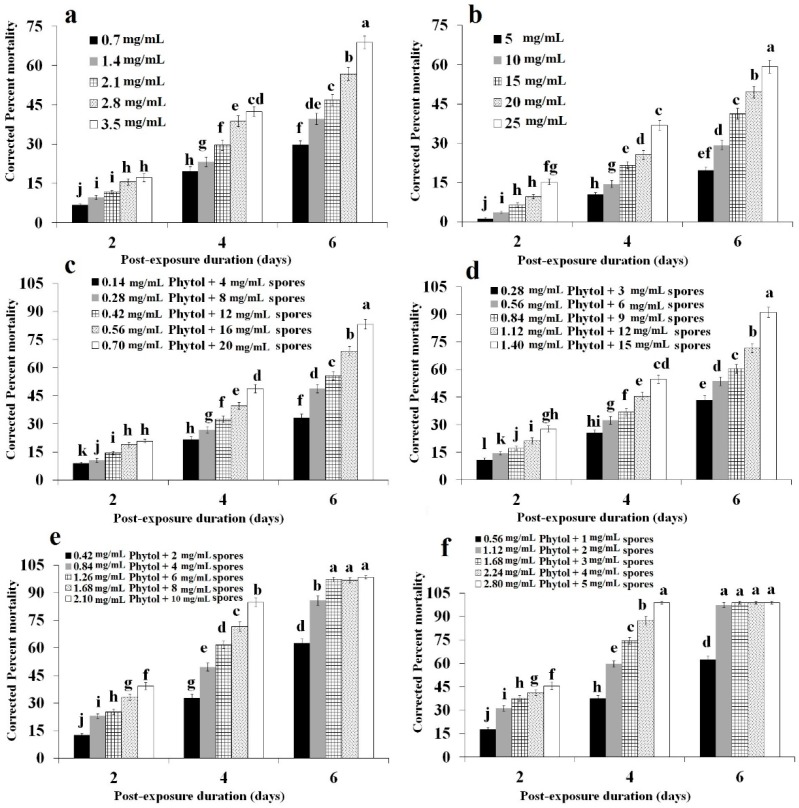
Corrected percent cumulative concentration-mortality response of *Oligonychus afrasiaticus* (McGregor) against (**a**) phytol; (**b**) spores of *Beauveria bassiana* B6903; (**c**) 20% phytol:80% spores; (**d**) 40% phytol:60% spores; (**e**) 60% phytol:40% spores; and (**f**) 80% phytol:20% spores. Bars (means ± SE) followed by different letter(s) are significantly different (Fisher’s LSD test, α = 0.05).

Toxicity of phytol and pathogenicity of the spores of *B. bassiana* B6903 calculated in terms of mortality varied significantly (Figure 2). Sole application of phytol or spores failed to impart 100% mortality within 6-days of post-application. Phytol at all tested concentrations (*F* = 559.95; df = 4, 48; *p* < 0.0001), time intervals (*F* = 119.88; df = 2, 48; *p* < 0.0001), and their interaction (*F* = 61.33; df = 8, 48; *p* < 0.0001) showed significant differences in mortality of old world date mites (Figure 2a). Spore application found to be the least pathogenic treatment also showed significant differences in mortality calculated after 2, 4, and 6 days post-infection time intervals (*F* = 134.74; df = 2, 48; *p* < 0.0001), concentrations (*F* = 968.06; df = 4, 48; *p* < 0.0001), and their interaction (*F* = 83.75; df = 8, 48; *p* < 0.0001) (Figure 2b).

Joint application of spores and phytol with different proportions significantly enhanced the treatment effect by imparting mortality among old world date mites (Figure 2c–f). Comparatively, treatments with higher proportions of phytol were found to be more potent compared with spores with higher proportions (Table 2). Overall, 80% phytol: 20% spores treatment found to be the most potent that tremendously reduced the LC_50_ value (1.62 mg/mL) compared with sole application of phytol (LC_50_ = 3.28 mg/mL) and spores (LC_50_ = 28.28 mg/mL). The high joint toxicity index (691) calculated based on their LC_50_ values designated this action as “synergistic” (Table 2). Concentration mortality response calculated after 2, 4, and 6 days post-exposure time intervals (*F* = 324.06; df = 2, 48; *p* < 0.0001), concentrations (*F* = 1582.51; df = 4, 48; *p* < 0.0001), and their interaction (*F* = 147.19; df = 8, 48; *p* < 0.0001) showed significant differences in mortality of old world date mites (Figure 2f). Furthermore, 60% phytol: 40% spores treatment, which imparted LC_50_ of 2.83 mg/mL led to a synergistic action (joint toxicity = 246). Mortality response of old world date mites at different concentrations (*F* = 1176.24; df = 4, 48; *p* < 0.0001), time intervals (*F* = 317.99; df = 2, 48; *p* < 0.0001), and their interaction (*F* = 73.92; df = 8, 48; *p* < 0.0001) showed significant differences (Figure 2e).

Concentration-mortality response bioassays with higher proportions of the spores of *B. bassiana* resulted in an “antagonistic” type of action in both cases including 20% phytol:80% spores (joint toxicity = 29.6), and 40% phytol:60% spores (joint toxicity = 60) as shown in Table 2. The mortality of *O. afrasiaticus* fed on date palm leaf disks treated with 20% phytol:80% spores calculated at different concentrations (*F* = 1306.47; df = 4, 48; *p* < 0.0001), time intervals (*F* = 161.02; df = 2, 48; *p* < 0.0001), and their interaction (*F* = 164.22; df = 8, 48; *p* < 0.0001) imparted significant differences (Figure 2c). Similarly, 40% phytol:60% spores treatment also showed significant differences in mortality of old world date mites calculated after 2, 4, and 6 days post-exposure time intervals (*F* = 143.65; df = 2, 48; *p* < 0.0001), concentrations (*F* = 1267.14; df = 4, 48; *p* < 0.0001), and their interaction (*F* = 104.87; df = 8, 48; *p* < 0.0001) as shown in Figure 2d.

### 2.3. Host Defense Related Enzyme Activities

**Figure 3:**
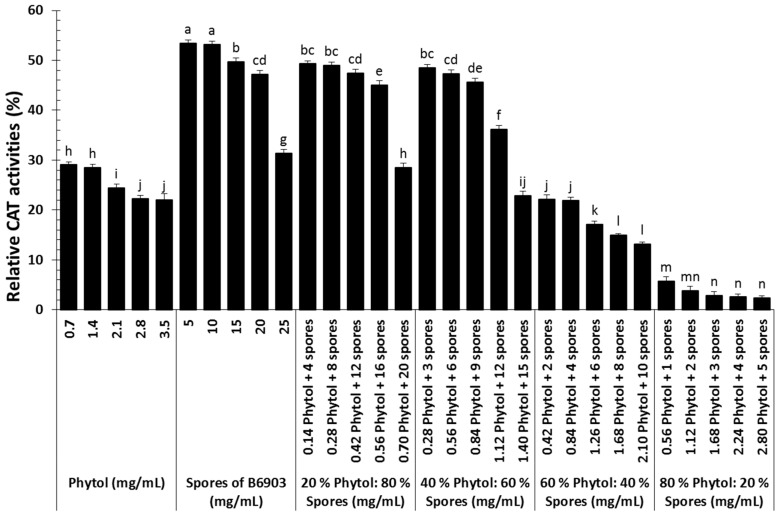
Catalase (CAT) activities (%) of *Oligonychus afrasiaticus* (McGregor) fed on date palm leaf disks treated with different combinations of phytol and spores of *Beauveria bassiana* B6903 relative to control. Bars (means ± SE) followed by different letter(s) are significantly different (Fisher’s LSD test, α = 0.05).

Toxicity of phytol and spores in different proportions induced different levels of antioxidant enzymes including catalase (CAT) and superoxide dismutase (SOD). Ninety-six hours after exposure, CAT activities of old world date mites differed significantly among different treatments (*F* = 2591.27; df = 5, 120; *p* < 0.0001), concentrations (*F* = 377.59; df = 4, 120; *p* < 0.0001), and their interaction (*F* = 33.56; df = 20, 120; *p* < 0.0001). Overall, the least potent treatment (spores of *B. bassiana* B6903) established in the current study showed the highest activities of CAT. However, different concentrations of the most potent treatment (80% phytol:20% spores) could not induce CAT activities and remained significantly at the lowest level compared to other treatments (Figure 3).

**Figure 4:**
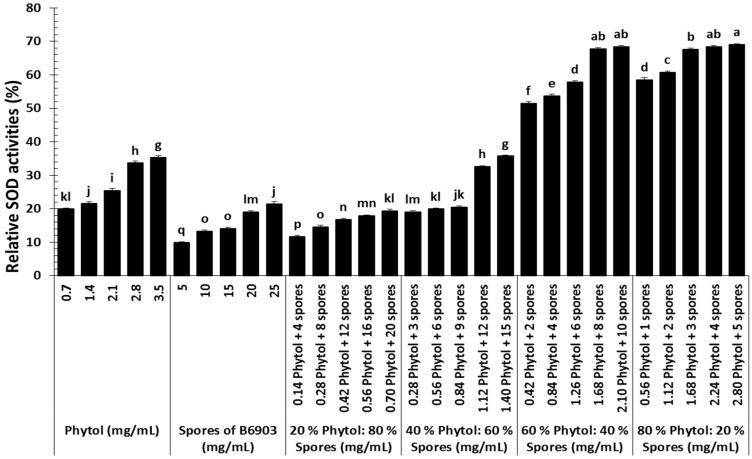
Superoxide dismutase (SOD) activities (%) of *Oligonychus afrasiaticus* (McGregor) fed on date palm leaf disks treated with different combinations of phytol and spores of *Beauveria bassiana* B6903 relative to control. Bars (means ± SE) followed by different letter(s) are significantly different (Fisher’s LSD test, α = 0.05).

The most potent treatment (80% phytol:20% spores) at its high concentration, established in the current study tremendously induced the SOD activities of old world date mites (Figure 4). The interaction of six treatments prepared by combining phytol and spores in different proportions with their different concentrations was significantly different (*F* = 30.39; df = 20, 120; *p* < 0.0001). Overall, spores and 20% phytol:80% spores treatment at all of their tested concentrations did not greatly induce SOD activities compared with the control.

**Figure 5:**
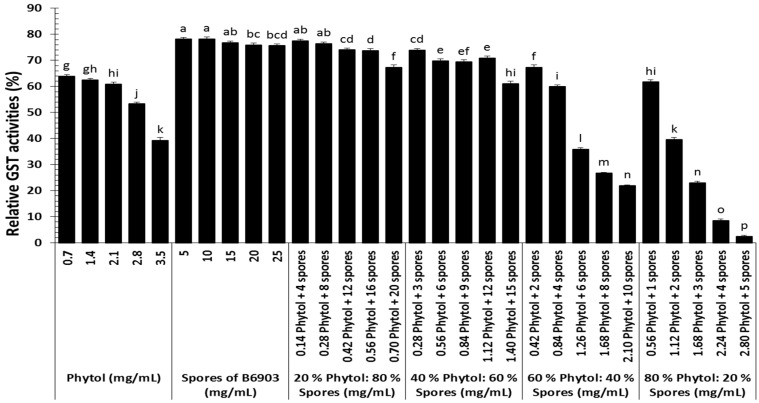
Glutathione S-transferase (GST) activities (%) of *Oligonychus afrasiaticus* (McGregor) fed on date palm leaf disks treated with different combinations of phytol and spores of *Beauveria bassiana* B6903 relative to the control. Bars (means ± SE) followed by different letter(s) are significantly different (Fisher’s LSD test, α = 0.05).

Lethality of different treatments induced different patterns of detoxification enzymes including Glutathione S-transferase (GST) and Acetylcholinesterase (AchE). Old world date mites upon exposure with different treatments (*F* = 3527.72; df = 5, 120; *p* < 0.0001), concentrations (*F* = 1140.52; df = 4, 120; *p* < 0.0001), and their interaction (*F* = 184.77; df = 20, 120; *p* < 0.0001), showed significant differences in their GST activities (Figure 5). Overall, the least potent treatments especially spores, 20% phytol:80% Spores, and 40% phytol:60% Spores relatively induced higher GST activities (>60%) compared with the most potent treatments having higher proportions of phytol. However, negligible relative GST activities (2.54%) were induced by the most potent treatment (80% phytol:20% spores) at its maximum concentration.

**Figure 6:**
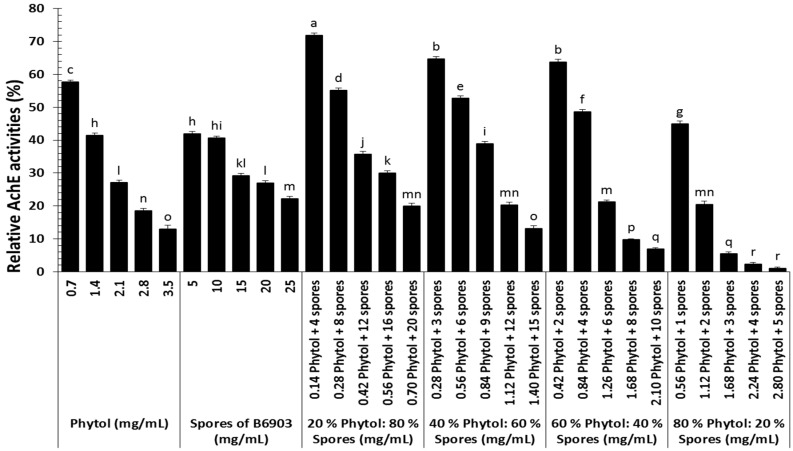
Acetylcholinesterase (AchE) activities (%) of *Oligonychus afrasiaticus* (McGregor) fed on date palm leaf disks treated with different combinations of phytol and spores of *Beauveria bassiana* B6903 relative to control. Bars (means ± SE) followed by different letter(s) are significantly different (Fisher’s LSD test, α = 0.05).

Enzymatic activities of AchE among old world date mites revealed significant differences among all treatments (*F* = 628.90; df = 5, 120; *p* < 0.0001), concentrations (*F* = 2921.52; df = 4, 120; *p* < 0.0001), and their interaction (*F* = 59.73; df = 20, 120; *p* < 0.0001). Overall, all the treatments at their lowest concentration showed their highest AchE activities (Figure 6). Each treatment AchE activities declined with the increase in concentration. Overall, the maximum reduction of AchE activities compared with the control were recorded from higher concentrations of the most potent treatment such as 80% phytol:20% spores.

## 3. Discussion

Pairing of sub-lethal concentrations of phytol with *Beauveria bassiana*, which established synergistic action in the current study, alter the defense mechanism of *Oligonychus afrasiaticus*. Complex changes in their enzymatic profile in response to joint application ultimately lead to enhance mortality response of old world date mites by reducing host killing time in comparison with either agent being used alone. This approach is ideal to circumvent slow mode of action criticism against entomopathogenic fungi as bio-control agent.

Screening the plant secondary metabolites as potential acaricide is much demanding study due to resistance development among these short life span and high reproductive potential mites. In this regard, we have specifically selected an eco-friendly compound, phytol that belongs to acyclic monounsaturated diterpene alcohol, which previously reported as antibacterial [33,34,35], antischistosomal [36,37], antioxidant [38], antinociceptive [38], and non-mutagenic food additive as fragrant [39]. However, phytol as acaricide has never been tried previously. Toxicity of phytol established in the current study ratified promising acaricidal potential against *O. afrasiaticus*. Concentration-mortality response bioassays revealed time and concentration-dependent lethality of phytol against old world date mites. In addition, we observed that the exposed old world date mites showed slow movements, which ultimately imparted mortality. However, we have recorded bit higher concentration of phytol to impart LC_50_ (3.28 mg/mL) for old world date mites. In the past, numerous studies successfully reduced the toxicant dose and mortality time by combining spores of entomopathogenic fungi [28,40,41]. Prior to record synergistic or antagonistic response of joint application, it is mandatory to calculate in vitro action of phytol against the spores of *B. bassiana*. In this study reported here, we observed that the growth parameters of *B. bassiana* B6903 were least affected by the action of phytol at all tested concentrations. The Biological Index computed from vegetative growth, percent germination and sporulation of *B. bassiana* ascertained compatibility with phytol. These in vitro compatibility results corroborate “Joint Application” to ascertain maximum treatment effect in terms of high mortality at low concentration under short period against old world date mites.

Interaction of entomopathogenic fungal spores with chemicals can have antagonistic, neutral or synergistic mortality response against target pest population [28,42]. Increased mortality of *O. afrasiaticus* with the use of phytol and entomopathogenic fungus *B. bassiana* in combinations was recorded in the current study. However, their combined application exhibited varying interactions such as Synergistic or Antagonistic. Among the four combinations, antagonistic response did occur at 20% phytol:80% Spores and 40% phytol:60% Spores with Joint toxicity co-efficient 29.6 and 60, respectively. Such interaction (Antagonistic) in these treatments might because of high proportion of *B. bassiana* spores. In addition, our results revealed that *B. bassiana* spores alone found to be the least pathogenic treatment and their combinations with lower phytol concentration was not enough for old world date mites to impart synergistic response. On the other hand, 60% phytol:40% Spores and 80% phytol:20% Spores combinations revealed synergistic interaction with Joint toxicity co-efficient 246 and 691, respectively (Table 2). These findings exhibited that addition of phytol into the suspension of *B. bassiana* spores significantly improved the treatment efficacy by enhancing mortality of *O. afrasiaticus*. In addition, we might suggest that 80% phytol:40% spores and 80% phytol:20% spores combinations with high “joint toxicity coefficients” and improved mortality response (98.40%) demonstrated the possible effectiveness of a joint application approach for the management of old world date mites. Numerous studies in the past have demonstrated similar mortality trends in their target pest species upon exposure with dual application methodology including fungal spores and pesticides. For instance, simultaneous application of the combination of chlorantraniliprole and *Metarhizium anisopliae* imparted both synergistic and antagonistic mortality response against *Locusta migratoria* (Meyen) [28]. Laboratory bioassays conducted against two spotted spider mites to check the compatibility of *Beauveria bassiana* with azadirachtin and flufenoxuron revealed synergistic interaction in case of *B. bassiana* with flufenoxuron. On the other hand, *Beauveria bassiana* with azadirachtin showed an additive effect against *Tetranychus urticae* Koch (Acari: Tetranychidae) [41]. These findings coincide with our results in that joint application improved the mortality of target pest species along with decline in concentration of chemical, which ultimately contributes in declining the incidence of pesticide resistance development in old world date mites.

Oxidative metabolism, a dominant source of energy leads to the generation of reactive oxygen species (ROS) such as superoxide anion (O_2_^●–^), and hydrogen peroxide (H_2_O_2_). The built-in antioxidant defense mechanism protects the biological systems by regulating ROS production. However, stressful situations disproportionate the target host intrinsic antioxidants and ROS balance by eliciting oxidative stress [22,43]. The target host has evolved accordingly antioxidant defense system to maintain homeostasis. The enzymatic activities of antioxidants measured in the current study showed moderate to complex interaction might be because of remarkable toxin-pathogen synergy.

The first-line antioxidant defense enzyme, SOD is indispensable for the catalysis of super oxide anion radicals into hydrogen peroxide. Our results showed tremendously high SOD activity in response to joint application with high proportions of phytol. The high up-regulation of SOD activities among the most lethal treatment is in line with previous studies conducted on the mechanism of antioxidant system in target host against pathogen infection [22,44]. The increased SOD activities in response to the most lethal treatments might result in its accumulation for the rapid elimination of ROS in order to safeguard the macromolecules. Their accumulation at lateral stages of infection was tremendously reduced resulting the failure of ROS elimination, which ultimately stop the target host cellular processes by denaturation of biomolecules [28,45].

Catalase is a ubiquitous ROS-scavenging enzyme that guards the target host from oxidative damage by taking part in H_2_O_2_ elimination [22]. The involvement of CAT in the target host as a component of antioxidant defense against pathogens and toxins is well-established from previous investigations [21,28]. The reduced enzymatic activities of CAT among the most lethal treatment reported in the current study might result in the removal of ROS at lower level. Such reductions in CAT activities could denature target host different biomolecules, ultimately heading the target host towards death. The reduced CAT activities in response to the most lethal treatment is in agreement with other studies on the role of host antioxidant defense against the joint application of chlorantraniliprole and *M. anisopliae* [28]. These findings enable us to suggest that variations in the activities of CAT in the same target host are mainly due to the lethality of the treatment.

Glutathione S-transferases is an important enzyme widely involved in the detoxification of harmful compounds [46]. In the current study, physiological responses of *O. afrasiaticus* stimulate the activities of enzymes involved in the detoxification of pathogen-toxin application by enhancing the metabolic mechanisms of old world date mites. We recorded enhanced activity of GST enzyme indicating that GST promptly detoxifies the treatments, especially sole applications of spores and toxin, and antagonistic combinations, such as 20% phytol:80% spores, and 40% phytol:60% spores. The enhanced GST activities in these combinations trigger the solubility of target compound, which ultimately promotes the degradation and excretion of compounds from the target host. Previous studies strengthened our findings and coincides with our results, which reported that lethality of the compound regulates the GST activities [47,48]. Furthermore, their findings suggested that less toxic compounds greatly induced target host GST activities. However, synergistic combinations established in the current study (especially 2.24 mg/mL phytol + 4 mg/mL spores and 2.80 mg/mL phytol + 5 mg/mL spores) found to be the most lethal treatments showed the reduced activity of GST. These findings are similar to the results of AlJabr et al. [47], who also reported similar GST activity of coumarin-fed red palm weevil larvae. Furthermore, their findings suggested that the most lethal treatment targeted the enzymatic activities of GST, which ultimately led towards the reduction in GST response by masking their enzymatic activities.

Acetylcholinesterase (AChE) is a serine hydrolase enzyme involved in the termination of nerve impulses by rapid hydrolysis of acetylcholine (neurotransmitter) [49]. However, present study demonstrated that the application of phytol or spores alone or in different proportions elicited concentration-dependent activities of AChE. Each treatment at their lower concentrations triggered the highest response in terms of AChE activities. In contrast, reduced activities of AChE recorded from the highest concentrations might be due to the accumulation of acetylcholine, which ultimately lead towards the dysfunction of the neuromuscular system of old world date mites. The current findings could further be explained in the light of Singh and Singh [50] and Zibaee [51], who reported restlessness, paralysis, and ataxia in the target host, which ultimately imparted mortality due to the inhibition of AChE activities.

## 4. Materials and Methods

### 4.1. Mite Collection and Maintenance

The bunches of date palm fruits infested with old world date mites were collected from Agricultural Research Station, Al-Ahsa 31982, Saudi Arabia (25.458626, 49.567936). The population of *Oligonychus afrasiaticus* was maintained under controlled conditions (25 ± 1 °C, 62.5 ± 12.5% RH, and a 16:8 h (L:D) photoperiod).

### 4.2. Entomopathogenic Fungus

The highly virulent isolate of *Beauveria bassiana* B6903 screened in the preliminary study (data not shown) procured from the USDA-ARS collection was used in this study against old world date mites. The isolate B6903 was grown on potato dextrose agar medium (Oxoid, Hampshire, UK) in complete darkness at 25 ± 0.5 °C. The required spore suspension of B6903 was prepared by Neubauer hemocytometer (Wertheim, Germany) in Tween 80 (0.05%) (Sigma Aldrich, London, UK; P4780-500 mL).

### 4.3. Plant Secondary Metabolite

Phytol, an important fraction of *Cucumis sativus* extract was selected in this study due to its toxicity against *O. afrasiaticus* determined in our preliminary study. Phytol was purchased from Sigma Aldrich, London, UK (catalogue no. 139912-10G). The required concentrations of phytol were prepared using environmental friendly dimethyl sulfoxide (DMSO) solvent.

### 4.4. Compatibility Bioassays

Pure culture of *B. bassiana* isolate B6903 was multiplied in Petri dishes (115 mm × 20 mm) provided with PDA in complete darkness at 25 ± 0.5 °C. The mature 21-day culture of B6903 was suspended in 0.05% Tween 80 to prepare 1 × 10^6^ spores/mL suspension using Neubauer hemocytometer. The compatibility of spore suspension with tested plant secondary metabolite (phytol) was determined using different concentrations (0.70, 1.40, 2.10, 2.80, and 3.50 mg/mL). Due to the hydrophobic nature of phytol, we used 1% DMSO to evenly solubilize the active ingredient. Spore compatibility with phytol was determined by the biological index (BI) by recording spore germination, vegetative growth, and sporulation [52].

#### 4.4.1. Impact of Phytol on the Fungal Vegetative Growth and Sporulation

Vegetative growth of *B. bassiana* isolate B6903 in the presence of different concentrations of phytol was assessed by poison food technique [22]. In brief, specific quantity of the phytol solution dissolved in DMSO was mixed with PDA to separately prepare each strength (0.70, 1.40, 2.10, 2.80 and 3.50 mg/mL) of fungal growth media. Phytol was added into the PDA after cooling the media to 50 °C in a conical flask. After mixing each concentration flask, each strength PDA was separately poured into seven Petri dishes (115 mm × 20 mm) to prepare seven independent replicates. PDA media for control treatment was provided with 1% DMSO. An aliquot of 5 µL of *B. bassiana* B6903 suspension at a concentration of 1 × 10^6^ spores/mL was pipetted to the center of each Petri dish. The inoculated plates after sealing with Parafilm were incubated at 25 ± 0.5 °C in complete darkness. After 12-days of incubation, vegetative growth of each experimental unit was determined by measuring the five perpendicular radial lengths (mm) of fungal growth. On the other hand, sporulation of each treatment was calculated by cutting 15 mm diameter sporulation colony of *B. bassiana* B6903 with the help of a sterile cork borer. Each disk after agitation in glass beaker was vortexed to count the number of spores in 0.05% Tween 80 solution under the compound microscope using Neubauer hemocytometer. One-way ANOVA analysis was performed for vegetative growth and sporulation. Significant differences in vegetative growth and sporulation in response to different concentrations of phytol were determined by Fisher’s LSD test (α = 0.05) [53].

#### 4.4.2. Impact of Phytol on the Fungal Spore Germination

Potato dextrose agar supplemented petri dishes (115 mm × 20 mm) prepared with different strength of phytol including 0.70, 1.40, 2.10, 2.80, and 3.50 mg/mL were prepared to determine their impact on the germination of spores of *B. bassiana* B6903. Each phytol strength Petri dish was separately inoculated with 200 µL spore suspension (1 × 10^6^ spores/mL) of *B. bassiana* B6903. In case of control, only 1% DMSO was incorporated into the PDA. Each experimental unit was incubated for 18 h at 25 ± 0.5 °C in complete darkness. Spores germination of B6903 in response to each tested concentration of phytol was determined by counting 100 spores from five different fields of vision under compound microscope. Seven replicates were prepared likewise for each concentration. The percent of spore germination data were analyzed by one way ANOVA. Significant differences in the percent of spore germination against different concentrations of phytol were determined by Fisher’s LSD test (α = 0.05) [53].

#### 4.4.3. Calculation of Compatibility of Phytol with Spore Suspension

Toxicological impacts of phytol on the growth of *B. bassiana* B6903 were categorized on the basis of the biological index [52]. BI values computed from the spore germination (GR), vegetative growth (VG), and sporulation (SP) data narrate whether the combination is compatible (BI > 66) or toxic (BI > 42). Prior to calculating the biological index, germination, sporulation, and vegetative growth data were corrected by their respective control. The biological index for *B. bassiana* B6903 in response to phytol was calculated using the following formula:

BI = [47 × VG + 43 × SP + 10 × GR]/100

### 4.5. Concentration Mortality Response of B. bassiana against O. afrasiaticus

Leaf-dip bioassay already used against old world date mites with some modifications was used to determine the pathogenicity of *B. bassiana* B6903 against old world date mites [6]. Date palm leaves were collected from those trees where no acaricides had been applied. Prior to dipping the leaf disks in each concentration of spore suspension (5, 10, 15, 20, and 25 mg/mL) prepared by weighing with an analytical balance, leaves were washed with distilled water. After dipping the leaf-disks in each concentration, disks were placed in Petri dishes (150 mm × 20 mm) provided with sterile water-soaked cotton in order to avoid leaf-disk desiccation. The edges of the leaf-disks were surrounded with cotton roll supplied with water. After drying the leaf-disks, 50 mites were transferred on each leaf-disk using a fine brush under microscope. Each replicate comprised of three leaf-disks arranged in one Petri dish. Five replicates were prepared likewise for each concentration. Each experimental unit was kept at 25 ± 1 °C, 62.5 ± 12.5% RH, and 16:8 h (L:D) photoperiod. Control treatment was prepared by dipping the leaf disks in 0.05% Tween 80 solution. The study was repeated over time in next season. The live and dead *O. afrasiaticus* on each leaf-disk were recorded after 24 h until 16-day post-exposure. The cadavers of old world date mites were transferred under a laminar airflow cabinet onto wet filter paper to promote fungal growth in order to make sure the actual cause of mortality. Natural mortality factor from treatment mortality was corrected by Abbott’s formula [54]. Mortality data were angularly-transformed. Concentration mortality response at specific time intervals was analyzed by repeated measures ANOVA and means by Fisher’s LSD test [53].

### 4.6. Concentration Mortality Response of Phytol against Oligonychus afrasiaticus

The toxicity of phytol was evaluated against *O. afrasiaticus* by leaf-dip bioassays. Date palm leaf-disks were dipped in different concentrations of phytol (0.70, 1.40, 2.10, 2.80, and 3.50 mg/mL). After drying and surrounding the leaf-disks edges with dampened cotton, 50 deutronymphs (2nd nymphal stage) of *O. afrasiaticus* mites were transferred on each treated leaf-disk by a fine brush under a microscope. Petri dishes (150 mm × 20 mm) were incubated at 25 ± 1 °C, 62.5 ± 12.5% RH, and 16:8 h (L:D) photoperiod. The control treatment was prepared by dipping the leaf disks in 1% DMSO. Each replicate comprised of three leaf-disks arranged in one Petri dish. Five replicates were prepared likewise for each treatment. Concentration mortality response of phytol against old world date mites was also repeated in next season. Mortality data were recorded after 24 h until 16-day post-exposure. In order to eliminate natural mortality, phytol treatment mortality data were corrected by Abbott’s formula [54]. In addition, mortality data were angularly transformed. Concentration mortality response of phytol at specific time intervals against *O. afrasiaticus* was analyzed by repeated measures ANOVA and means by Fisher’s LSD test [53].

### 4.7. Synergism Bioassays

The possible synergism or antagonism of phytol with *Beauveria bassiana* B6903 was determined by combining them in different proportions (Table 3). After dipping leaf-disks in each treatment, fifty deutronymphs were confined in wet cotton surrounded leaf-disk arena in Petri dishes. Each replicate comprised of three leaf-disks arranged in a single Petri dish (150 mm × 20 mm). Five replicates for each combination were tested. The control treatment was prepared by dipping leaf-disks in 1% DMSO solution prepared in 0.05% Tween 80. After every 24 h of incubation at 25 ± 1 °C, 62.5 ± 12.5% RH, and a 16:8 h (L:D) photoperiod, live and dead mites were counted for 16-days under microscope. Each experimental unit mortality data were subjected to Probit analysis for LC_50_ determination [55]. Each treatment angularly transformed mortality data were subjected to analyze by repeated measures ANOVA and Fisher’s LSD test (α = 0.05) [53]. The joint application of phytol and suspension in different proportions led to synergistic or antagonistic interaction was determined using following Joint Toxicity equations [32]. Phytol was selected as standard agent.

Joint Toxicity = Actual toxicity index (T.I) of a mixture (M)/Theoretical toxicity index (T.I) of a mixture × 100

Actual T.I of a mixture = LC_50_ of standard agent/LC_50_ of mixture (M)

Theoretical T.I of a mixture = (T.I of agent a × % of agent a in M) + (T.I of agent b × % of agent b in M)

T.I = LC_50_ of standard agent/LC_50_ test sample

### 4.8. Exploration of Host Defense Related Enzymes Regulation

The activities of enzymes involved in host defense mechanism including detoxification (GST and AChE) and antioxidant (CAT and SOD) were calculated using standard methodologies provided by the manufacturer. Old world date mites treated by leaf-disk method with different concentrations of phytol (0.70, 1.40, 2.10, 2.80, and 3.50 mg/mL), *B. bassiana* B6903 (5, 10, 15, 20, and 25 mg/mL), and their joint application in different proportions (Table 3) were incubated at 25 ± 1 °C, 62.5 ± 12.5% RH, and 16:8 h (L:D). Mites of each experimental unit were separately homogenized in ice-cold 0.05 M Phosphate-buffered saline pH 7.0 supplemented with aprotinin (final concentration 1.5 µmol/L) and phenylthiourea (final concentration 20 µmol/L). Each sample after homogenization was centrifuged at 12,000× *g* at 4 °C for 15 min. The supernatant of each sample was used for protein quantification by standard methodology [56]. The enzymatic activities of SOD (Sigma Aldrich, catalogue no. 19160-1KT-F, London, UK), CAT (Sigma Aldrich, catalogue no. CAT100-1KT, London, UK), GST (Sigma Aldrich, catalogue no. CS0410-1KT, London, UK) and acetylcholinesterase (Abcam, catalogue no. ab138871, Shanghai, China) were calculated using their standard methodology protocols provided by the manufacturer. Each enzyme data were separately analyzed by two factor factorial analysis and Fisher’s LSD test (α = 0.05) [57].

## 5. Conclusions

In conclusion, synergistic and antagonistic effects vary with the proportion of phytol in combination with *B. bassiana* spores against old world date mites, *O. afrasiaticus*. The synergistic combination of fungal spore application with phytol exhibit an effective strategy for the management of old world date mites by lowering the dose of the toxin and pathogen. Additionally, this approach will assist the development of eco-friendly protection of date palm fruit in the context of IPM for long-term sustainable management of old world date mites.

## Figures and Tables

**Table 1:** Biological index-based classification of the toxicological impacts of various concentrations of phytol on *B. bassiana* B6903.

Treatments	Biological Index	Classification *
0.7 mg/mL	96.93	Compatible
1.4 mg/mL	94.39	Compatible
2.1 mg/mL	91.61	Compatible
2.8 mg/mL	89.24	Compatible
3.5 mg/mL	87.78	Compatible

* Classification criterion: compatible (BI > 66); moderately toxic (BI between 42 and 66); toxic (BI ≤ 42).

**Table 2:** Joint toxicity evaluation of phytol and *Beauveria bassiana* isolate B6903 against *Oligonychus afrasiaticus* (McGregor).

Treatment	LC_50_ (mg/mL)	Joint Toxicity	Type of Action *
**20% Phytol:80% Spores**	13.39 (11.00–16.29)	29.6	Antagonistic
0.14 mg/mL Phytol + 4 mg/mL spores			
0.28 mg/mL Phytol + 8 mg/mL spores			
0.42 mg/mL Phytol + 12 mg/mL spores			
0.56 mg/mL Phytol + 16 mg/mL spores			
0.70 mg/mL Phytol + 20 mg/mL spores			
**40% Phytol:60% Spores**	8.45 (6.94–10.26)	60	Antagonistic
0.28 mg/mL Phytol + 3 mg/mL spores			
0.56 mg/mL Phytol + 6 mg/mL spores			
0.84 mg/mL Phytol + 9 mg/mL spores			
1.12 mg/mL Phytol + 12 mg/mL spores			
1.40 mg/mL Phytol + 15 mg/mL spores			
**60% Phytol:40% Spores**	2.83 (2.41–3.33)	246	Synergistic
0.42 mg/mL Phytol + 2 mg/mL spores			
0.84 mg/mL Phytol + 4 mg/mL spores			
1.26 mg/mL Phytol + 6 mg/mL spores			
1.68 mg/mL Phytol + 8 mg/mL spores			
2.10 mg/mL Phytol + 10 mg/mL spores			
**80% Phytol:20% Spores**	1.62 (0.84–3.12)	691	Synergistic
0.56 mg/mL Phytol + 1 mg/mL spores			
1.12 mg/mL Phytol + 2 mg/mL spores			
1.68 mg/mL Phytol + 3 mg/mL spores			
2.24 mg/mL Phytol + 4 mg/mL spores			
2.80 mg/mL Phytol + 5 mg/mL spores			

* Type of action criterion: antagonistic (joint toxicity < 100); synergistic (joint toxicity ≥ 100) [32].

**Table 3:** Various concentrations of phytol and *Beauveria bassiana* isolate B6903 suspensions alone and in different proportions tested against *Oligonychus afrasiaticus.*

Phytol (mg/mL)	20% Phytol:80% Spores		40% Phytol:60% Spores		60% Phytol:40% Spores		80% Phytol:20% Spores		B6903 (mg/mL)
	Phytol (mg/mL)	B6903 (mg/mL)	Phytol (mg/mL)	B6903 (mg/mL)	Phytol (mg/mL)	B6903 (mg/mL)	Phytol (mg/mL)	B6903 (mg/mL)	
0.7	0.14	4	0.28	3	0.42	2	0.56	1	5
1.4	0.28	8	0.56	6	0.84	4	1.12	2	10
2.1	0.42	12	0.84	9	1.26	6	1.68	3	15
2.8	0.56	16	1.12	12	1.68	8	2.24	4	20
3.5	0.70	20	1.40	15	2.10	10	2.80	5	25
